# Early intestinal development of chickens divergently selected for high or low 8-wk body weight and a commercial broiler

**DOI:** 10.1016/j.psj.2024.103538

**Published:** 2024-02-08

**Authors:** Sydney R. Kinstler, Sara E. Cloft, Paul B. Siegel, Christa F. Honaker, John J. Maurer, Eric A. Wong

**Affiliations:** School of Animal Sciences, Virginia Tech, Blacksburg, VA 24061, USA

**Keywords:** genetic selection, intestinal morphology, Olfactomedin-4, PepT1, Mucin 2

## Abstract

The early posthatch period is crucial to intestinal development, shaping long-term growth, metabolism, and health of the chick. The objective of this study was to determine the effect of genetic selection on morphological characteristics and gene expression during early intestinal development. Populations of White Plymouth Rocks have been selected for high weight (**HWS**) and low weight (**LWS**) for over 63 generations, and some LWS display symptoms of anorexia. Intestinal structure and function of these populations were compared to a commercial broiler Cobb 500 (**Cobb**) during the perihatch period. Egg weights, yolk-free embryo BW, yolk weights, and jejunal samples from HWS, LWS, and Cobb were collected on embryonic day (**e**) 17, e19, day of hatch, day (**d**) 3, d5, and d7 posthatch for histology and gene expression analysis. The RNAscope *in-situ* hybridization method was used to localize expression of the stem cell marker, olfactomedin 4 (**Olfm4**). Villus height (**VH**), crypt depth (**CD**), and VH/CD were measured from Olfm4 stained images using ImageJ. mRNA abundance for Olfm4, stem cell marker Lgr5, peptide transporter PepT1, goblet cell marker Muc2, marker of proliferation Ki67, and antimicrobial peptide LEAP2 were examined. Two-factor ANOVA was performed for measurements and Turkey's HSD was used for mean separation when appropriate. Cobb were heaviest and LWS the lightest (*P* < 0.01). at each timepoint. VH increased in Cobb and CD increased in HWS compared to LWS (*P* < 0.01). PepT1 mRNA was upregulated in LWS (*P* < 0.01), and Muc2 mRNA was decreased in both HWS and LWS compared to Cobb (*P* < 0.01). Selection for high or low 8-wk body weight has caused differences in intestinal gene expression and morphology when compared to a commercial broiler.

## INTRODUCTION

Improvements to genetics, nutrition, husbandry, and health have increased the growth-rate of broiler chickens to produce the efficient meat birds used in the industry today. Substantial progress has been made in understanding the influence of genetics on metabolic mechanisms and disorders associated with selection of fast-growing chicks (Siegel, 2014; Siegel et al., 2019; Hartcher and Lum, 2020; Soglia et al., 2021; Siegel, 2023). An area lacking in information is the influence of genetic selection on intestinal developmental functions that shape structure and epithelial composition of the newly hatched chick. Studying these conditions could reveal information on developmental pathways and morphological characteristics that influence gut health and efficiency that are conserved or influenced in genetic selection.

The early posthatch period is the most critical period in the life of broiler chickens characterized by substantial physical and functional development of the gastrointestinal tract (Noy and Sklan, 1997; Uni et al., 2000; Lilburn and Loeffler, 2015; Kpodo and Proszkowiec-Weglarz, 2023). Maturation that occurs during this time period influences active immunity that shapes long term-growth, metabolism, and health (Iji et al., 2001; Wu et al., 2004). It is well documented that genetics influence nutrient requirements that are attributed to digestive and absorptive capabilities at the intestinal level (NRC, 1994). Changes to organ growth and digestive enzymes that aid in nutrient breakdown and absorption have been reported in chicks divergently selected for body weight (Nitsan et al., 1991). Postabsorptive utilization of nutrients may be most significant during the first few days of life as the metabolism shifts to digestion of exogenous feed (Jha et al., 2019). During this period, expression of nutrient transporters, stem cell proliferation and differentiation markers, and secretory cell functions that shape intestinal structure and function begins. Genetic selection for growth performance may affect differential gene expression that contributes to efficiency and health.

In the chicken small intestine, the epithelial structure consists of villi, finger-like projections that extend into the lumen of the intestine to absorb passing nutrients, and crypts, invaginations of intestinal tissue into the lamina propria (Uni et al. 2000). Stem cells are localized to the crypts and migrate up the villi, differentiating into absorptive and secretory cell types (Zhang et al., 2019). Olfactomedin 4 (**Olfm4**) is a robust stem cell marker that is strictly expressed in the intestinal crypt (Schuijers et al., 2014). Olfm4 is a secreted glycoprotein that plays a role in mucosal defense (Gersemann et al., 2012). Leucine-rich repeat containing G protein-coupled receptor 5 (**Lgr5**) is another intestinal stem cell marker involved in Wnt signaling for differentiation of stem cells towards secretory cells (Haegebarth and Clevers, 2009). Olfm4 mRNA is expressed greater than Lgr5 mRNA in the chicken intestine, possibly due to its role in the intestinal mucosa (Zhang and Wong, 2018). During embryonic chick development, villi are sculpted by embryonic day (**e**) 16, and oral consumption of the amniotic fluid at e17 promotes rapid development of the intestinal mucosa. Although intestinal maturation occurs rapidly in ovo, intestinal absorption of nutrients may be of minimal use (Kpodo and Proszkowiec-Weglarz, 2023). The significance of early establishment of digestive and absorptive functions before the onset of exogenous feeding prepares the chick for posthatch conditions (Sklan, 2001).

Growth and metabolism are mostly determined by processes that occur during embryonic development. A 14-to-50-fold increase in peptide transporter (**PepT1**) mRNA levels was observed from e16 to day of hatch (**doh**) (Chen et al., 2005). During hatching and in the first week posthatch, morphological changes and increased expression of enzymes and nutrient transporters begin storing amniotic substrates as nutritional reserves (Givisiez et al., 2020). Villus volume does not change within the first 2 d, but begins increasing rapidly at d3 (Uni et al., 1998). At 5 d posthatch, broiler chickens have been shown to maximize relative growth rate at 20% with maximum relative weight of the small intestine (Nir et al., 1993). Jejunal villus and crypt development occurs rapidly on d4 and 5 following hatches with most epithelial cells proliferating (Uni et al., 2000). By d7, the yolk residue is almost completely utilized, peak intestinal weight is achieved, and cell proliferation is maximized (van der Wagt et al., 2020). Chicken liver-enriched antimicrobial peptide 2 (**LEAP2**) is expressed in the chicken small intestine as an antimicrobial peptide contributing to the innate immune response and feed-intake regulation (Su and Wong, 2018, Zheng et al., 2022).

Long-term divergent selection for 8-wk body weight in chickens from a common founder population has resulted in low weight select (**LWS**) and high weight select **(HWS**) populations that display anorexic or compulsive overeating behaviors, respectively (Zelenka et al., 1988). Low weight select restrict feed intake, increase fatty acid oxidation in abdominal fat and possess greater metabolic flexibility and a lower threshold to anorexigenic factors (Cline et al., 2008; Dunnington et al., 2013; Zhang et al., 2014; Xiao et al., 2019). HWS have served as an obesity model as compulsive eaters and have been reported to increase feed intake with neuropeptide Y that contributes to intake stimulation (Newmyer et al., 2013). Early gastrointestinal development of these divergent populations under genetic selection of a single trait intensifies anatomical and endocrine factors that can be influenced by selection. Comparing these populations to a commercial broiler that has been selected for multiple traits would provide more background into the genetic potential for intestinal developmental functions during the perihatch period. The objective of this study was to investigate the influence of genetics on morphology and gene expression during early intestinal development of a commercial broiler and 2 populations selected for low and high 8-wk body weight.

## MATERIALS AND METHODS

### Animals and Housing

All animal protocols were approved by the Institutional Animal Care and Use Committee at Virginia Tech. LWS and HWS populations were established from a long-term divergent selection experiment for low or high body weight at 8 wk of age (Siegel, 1962). The founder population consisted of crosses of 7 inbred lines of White Plymouth Rocks with LWS and HWS selected lines developed and maintained as closed pedigree populations by continuous selection for low or high body weight at 56 d of age. Descriptions of breeding and maintenance of populations are described elsewhere (Siegel, 1962; Dunnington and Siegel, 1996; Márquez et al., 2010; Harrison et al., 2023). Cobb 500 (**Cobb**) were used for comparison of a commercial broiler with the LWS and HWS. All selected chickens were of random sex. Eggs from HWS, LWS, and Cobb were incubated at 37.5°C and 55% relative humidity. On doh, HWS and LWS were placed into single pens separating the populations. Cobb were received from a local hatchery and placed into battery cages (n = 20/cage). Feed in mash form (20% CP and 2,685 kcal ME/kg) and water were allowed *ad libitum*.

### Tissue Collection

On e17 and e19, 6 eggs from HWS and LWS populations and 8 Cobb eggs were randomly selected for sampling. Fewer HWS and LWS embryos were used due to availability and hatchability issues in these populations and to ensure that enough LWS and HWS chicks were available for posthatch analysis. Embryos were euthanized by cervical dislocation. Whole sections of jejunum were collected and fixed in neutral buffered formalin for 16 to 20 h at room temperature, 70% ethanol for 24 h at room temperature, and then stored in fresh 70% ethanol at 4°C. On doh, the number of hatched chicks set the number to be analyzed (7 Cobb and 8 HWS and LWS chicks) on doh (after hatch but before feeding), 1.5, 3, 5, and 7 d posthatch. Egg weights, yolk-free embryo BW, and yolk weights were recorded. Sections of jejunum were mounted in paraffin blocks for histology (StageBio, Mount Jackson, VA). Embedded tissues of 4 embryos or chicks from each population per timepoint were sectioned (6 μm) using a microtome and mounted onto Superfrost Plus microscope slides (Electron Microscopy Sciences, Hatfield, PA) for in situ hybridization (**ISH**). The remainder of the jejunum sections were flash frozen in liquid nitrogen for qPCR analysis.

### Intestinal Morphology

To examine intestinal morphology, ISH using the RNAscope 2.5 HD Assay-Brown detection kit (Advanced Cell Diagnostics, Newark, CA) was performed on sections of jejunum to localize gene expression (n = 4 chicks/population). A probe for stem cell marker Olfm4 (Zhang and Wong, 2018) was used to identify stem cells in the crypt. Slides were counterstained with 50% Gill's hematoxylin no. 1 (Sigma-Aldrich, St. Louis, MO) and placed in 0.02% ammonia water to turn the purple stain to blue. Sections were sealed with VectaMount (Biocare Medical, LLC, Pacheco, CA) and a glass coverslip. Images were captured using a Nikon Eclipse 80i microscope and DS-Ri2 camera (Nikon Instruments Inc., Melville, NY). Brightfield microscopy images were used to measure intestinal morphology parameters including villus height (**VH**) and crypt depth (**CD**) using ImageJ (National Institute of Health, Bethesda, MD). A minimum of 10 villi and 20 crypts were measured from 3 to 5 sections from each chick. CD was measured from the base of the crypt to the end of Olfm4 staining along the villi. VH was measured from the end of the crypt to the tip of the villi. The VH/CD ratio was calculated based on these measurements.

### Quantitative Real-Time PCR

Gene expression was analyzed using qRT-PCR. Total RNA was extracted from the jejunum of 6 to 8 chicks per population using the Direct-zol RNA MiniPrep kit (Zymo Research, Irvine, CA). RNA concentration was determined using a Nanodrop 1000 spectrophotometer (Thermo Fisher Scientific, Waltham, MA). One mg of total RNA was used for synthesis of complementary DNA using the High-Capacity cDNA Reverse Transcription kit (Applied Biosystems, Waltham, MA) qPCR reactions included 1 μL of forward primer (5 μM), 1 μL of reverse primer (5 μM), 5 μL Fast SYBR green master mix (Applied Biosystems), 1.5 μL sterile deionized water, and 1.5 μL of diluted cDNA (1:30). The cycling conditions were as follows: 95°C for 20 s, then 40 cycles of 90°C for 3 s and 60°C for 30 s. Primers were designed using Primer Express 3.0 (Applied Biosystems) and are listed in Table 1. Reactions were run in duplicate using an Applied Biosystems 7500 Fast Real-Time PCR system to examine expression of Olfm4, Lgr5, PepT1, goblet cell marker Mucin2 (**Muc2**), cell proliferation marker Ki67, and LEAP2. The geometric mean of ribosomal protein lateral stalk subunit P0 (**RPLP0**) and β-actin was used as a composite reference gene to even out any variable expression of a single reference gene (Vandesompele et al., 2002). The 2^−ΔΔCt^ method was used to determine fold change in gene expression using the ΔCt value of Cobb at e17 as the calibrator (Livak and Schmittgen, 2001).

**Table 1:** Primers for quantitative PCR.

Gene Name	Forward/Reverse Primers (5’ to 3)	Amplicon Size (bp)	Acc. No.
Olfactomedin 4 (Olfm4)	CAGGGCATATTTGCACAGGC/ GAACTTTGGGGAGGTGTGCT	72	NM_001040463.1
Leucine-rich repeat containing G protein-coupled receptor 5 (Lgr5)	TGGTTTGACCTTCGTTTGCAGGACA/TATACAATGGAGATCTGAAAACT	67	XM_425441.7
Peptide transporter 1 (PepT1)	CCCCTGAGGAGGATCACTGTT/ CAAAAGAGCAGCAGCAACGA	66	KF366603.1
Mucin 2 (Muc2)	CTGATTGTCACTCACGCCTTAATC/ GCCGGCCACCTGCAT	147	JX284122.1
Marker of Proliferation mKi67 (Ki67)	CACAGGCAAAGGCTGTCAAA/ TCCGTGCAATTTTCCTTGCT	63	NM_205322.1
Liver-enriched antimicrobial peptide 2 (LEAP2)	CTCAGCCAGGTGTACTGTGCTT/ CGTCATCCGCTTCAGTCTCA	66	NM_001001606.1
Ribosomal protein large subunit P0 (RPLP0)	GCGATTGCTCCCTGTGATG/ TCTCAGGTCCGAGACCAGTGT	58	NM_204987.2
β-actin	GTCCACCGCAAATGCTTCTAA/ TGCGCATTTATGGGTTTTGTT	78	NM_205518.1

### Statistical Analysis

Data distribution was assessed prior to analysis using JMP Pro v16.0 software. When found to be different from normal distribution a Box-Cox transformation was applied. Two-factor ANOVA considering genetic population and age were conducted and when *P* < 0.05 was achieved, mean separation was conducted using Tukey HSD test. When data were transformed, reported results are back-transformed values. Statistical significance was considered to be *P* < 0.05 for all tests.

## RESULTS

### Body Weight and Yolk Weight of Chicken Populations Divergently Selected for Body Weight Compared to a Commercial Broiler

**Figure 1:**
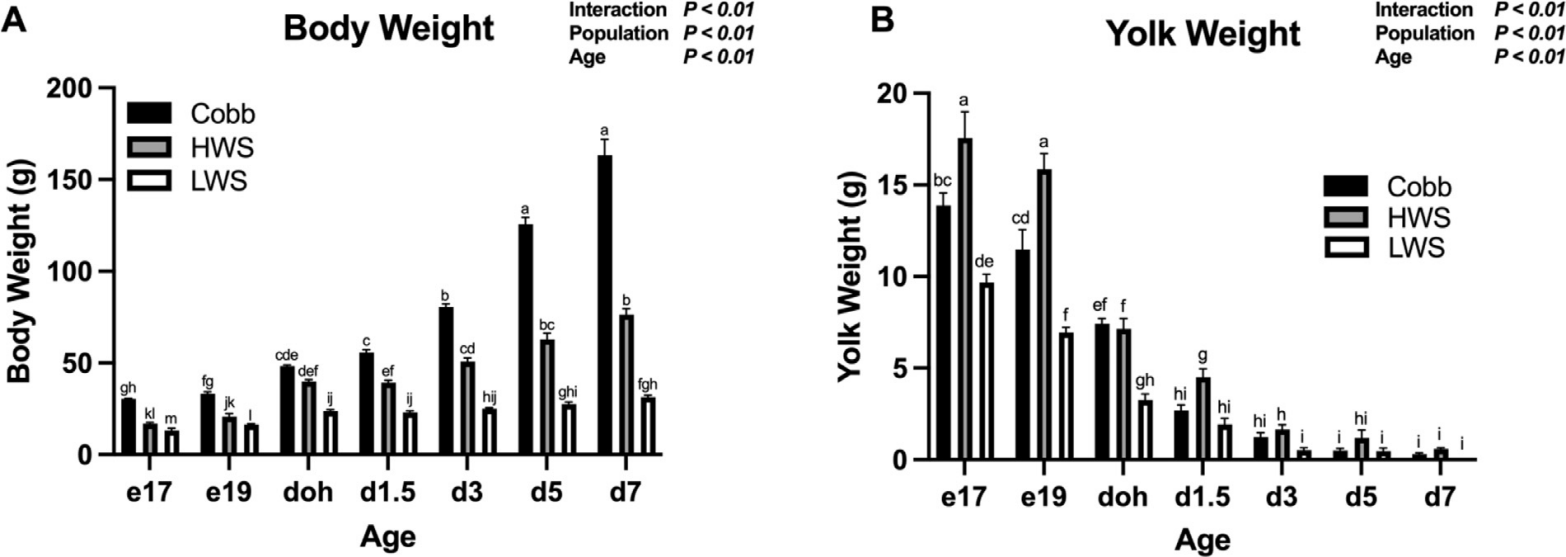
Body weight and yolk weight of chicken populations divergently selected for 8-wk body weight compared to a commercial broiler. Populations included Cobb500 (**Cobb**), high weight select (**HWS**), and low weight select (**LWS**). Yolk-free body weights (A) were measured on embryonic d 17 (e17) and embryonic d 19 (e19) and body weights were measured on day of hatch (**doh**), d 1.5 (d1.5), 3 (d3), 5 (d5) and 7 (d7) posthatch. Yolk weights (B) were measured from e17 to d7. Statistical significance was determined using 2-factor ANOVA and Tukey HSD mean separation. Each value represents the back-transformed means from 6 to 8 embryos or chicks. ^a-l^Bars with different letters are significantly different (*P* < 0.05).

For body weight, a Population × Age interaction was observed (*P* < 0.01; Figure 1A). During the embryonic period, Cobb were heavier than HWS at e17 (*P* < 0.0001) and e19 (*P* = 0.0002) which were heavier than LWS (*P* < 0.0001). At doh, Cobb and HWS were similar in weight (*P* = 0.35) and greater than LWS (*P* < 0.0001). From d1.5 to d7, Cobb were heavier than HWS (*P* < 0.0001), which in turn were heavier than LWS (*P* < 0.0001). By d7, Cobb were over twice the weight of HWS and 4-fold heavier than LWS.

For yolk weight, a Population × Age interaction was observed (*P* < 0.01; Figure 1B). Yolk weights were greater in HWS than Cobb on e17 (*P* = 0.0009) and e19 (*P* < 0.0001), which were heavier than LWS (*P* < 0.05)*.* At doh, HWS and Cobb had similar yolk weights (*P* = 0.99), which were both heavier than LWS yolk weights (*P* < 0.0001). At d1.5, HWS yolk weights were heavier than both Cobb (*P* = 0.046) and LWS (*P* = 0.012). At d 3, yolk weights of HWS were greater than LWS (*P* = 0.0039). No differences among populations were observed at d5 (*P* = 0.31) or d7 (*P* = 0.76).

### Morphology Parameters of Chicken Populations Divergently Selected for Body Weight Compared to a Commercial Broiler

**Figure 2:**
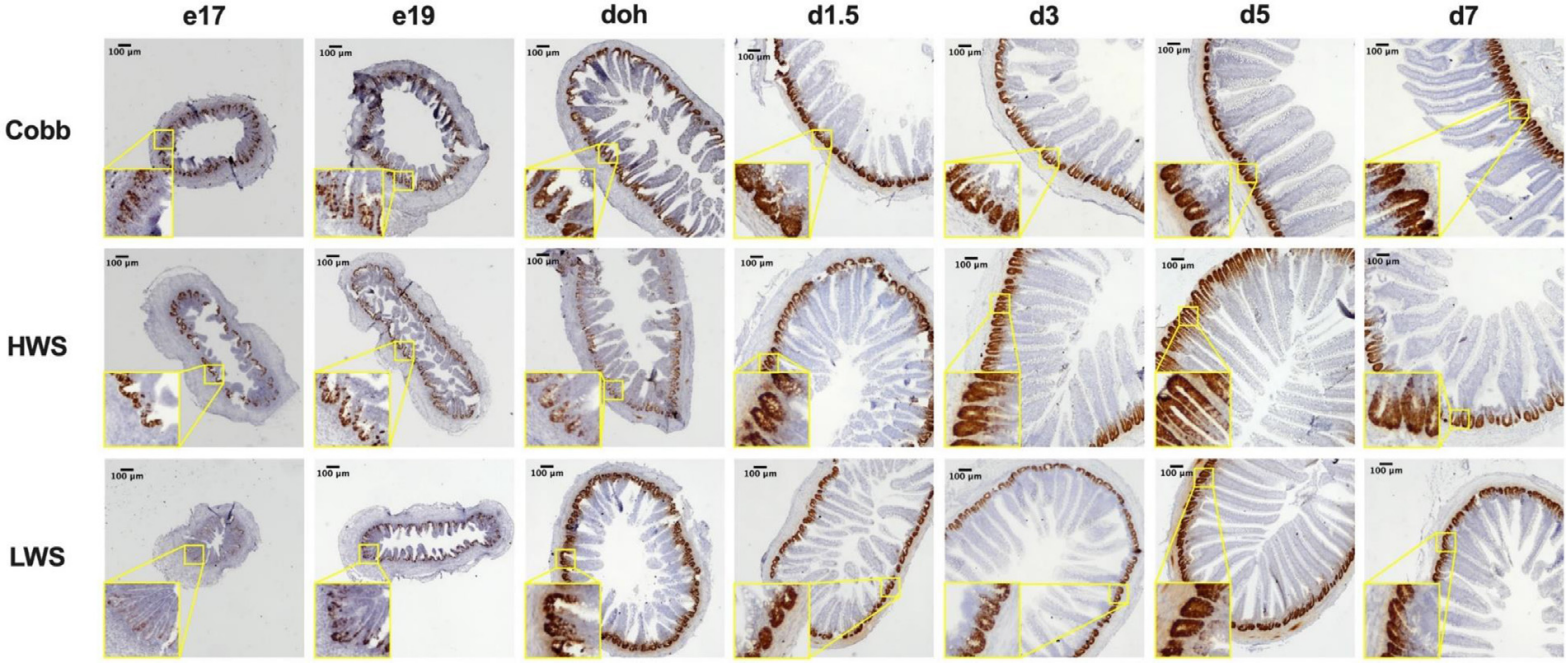
In-situ hybridization of stem cell marker Olfm4 in the jejunum of chicken populations divergently selected for 8-wk body weights compared to a commercial broiler. Populations included Cobb500 (**Cobb**), high weight select (**HWS**), and low weight select (**LWS**). In situ hybridization was performed on embryonic d 17 (e17), embryonic d 19 (e19), day of hatch (**doh**), d 1.5 (d1.5), 3 (d3), 5 (d5) and 7 (d7) posthatch. Expression of Olfm4 mRNA was revealed as the brown staining localized to intestinal crypts where stem cells reside. Sections were counterstained with hematoxylin. Images were captured at 40X magnification. Insets show magnified images of selected crypts.

Crypt depth and VH were measured after staining for the stem cell marker Olfm4 (Figure 2). There was robust staining for Olfm4 mRNA in the crypts of Cobb, HWS and LWS at all embryonic and posthatch ages, with the exception of LWS at e17, which showed only light staining for Olfm4. This may indicate a delayed development of stem cells in LWS.

**Figure 3:**
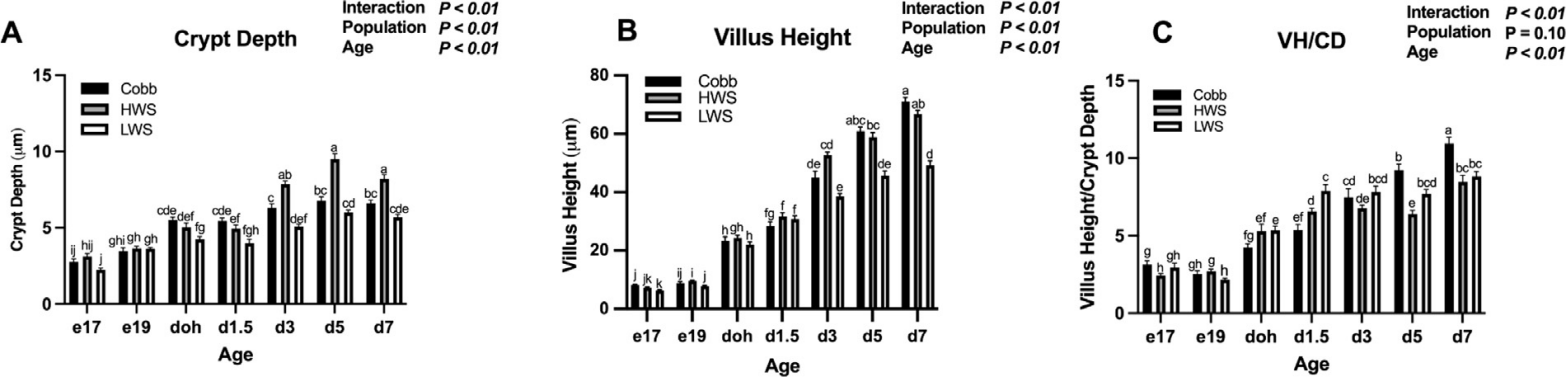
Morphological parameters of chicken populations divergently selected for 8-wk body weights compared to a commercial broiler. Populations included Cobb500 (**Cobb**), high weight select (**HWS**), and low weight select (**LWS**). Expression of stem cell marker Olfm4 mRNA was used to localize stem cells in the crypt of the jejunum using in situ hybridization to measure (A) crypt depth (**CD**) and (B) villus height (VH) to calculate the (C) villus height/crypt depth ratio (**VH/CD**). Measurements of morphology and body weight occurred on embryonic d 17 (e17), embryonic d 19 (e19), day of hatch (doh), d 1.5 (d1.5), 3 (d3), 5 (d5) and 7 (d7) posthatch. Statistical significance was determined using 2-factor ANOVA and Tukey HSD mean separation. Each value represents the back-transformed means from 6 to 8 embryos or chicks. ^a-k^Bars with different letters are significantly different (*P* < 0.05).

For CD, a Population × Age interaction was observed (*P* < 0.01; Figure 3A). There was no difference in CD for all populations on e17 (*P* = 0.095) and e19 (*P* = 0.65). CD for Cobb was greater than LWS on doh (*P* = 0.022) and d1.5 (*P* = 0.046), while on d3 to d7, CD for HWS was greater than Cobb (*P* < 0.0001) and LWS (*P* < 0.0001).

There was a Population × Age interaction for VH (*P* < 0.01; Figure 3B). At e17, VH of Cobb was greater than LWS (*P* = 0.0058), while at e19, VH of HWS was greater than LWS (*P* = 0.0004). There was no difference in VH among the 3 populations at doh (*P* = 0.33) and d1.5 (*P* = 0.19). At d3, VH of HWS was greater than that of LWS (*P* < 0.0001). On d5 and d7, VH of both Cobb and HWS were greater than LWS (*P* < 0.0001).

The VH/CD ratio also showed a Population × Age interaction (*P* < 0.01; Figure 3C). At e17, VH/CD of Cobb was greater than HWS (*P* = 0.0095), while at e19, VH/CD of HWS was greater than LWS (*P* = 0.011). At doh VH/CD of LWS was greater than Cobb (*P* = 0.015). At d1.5, VH/CD of LWS was greater than both Cobb (*P* < 0.0001) and HWS (*P* = 0.012). At d3, there was no difference in VH/CD among the 3 populations (*P* = 0.26). At d5, VH/CD of Cobb was greater than HWS (*P* < 0.0001), while at d7, VH/CD of Cobb was greater than both HWS (*P* = 0.0009) and LWS (*P* = 0.0014).

### Gene Expression of Stem Cells, Enterocytes, and Secretory Cells in the Jejunum of Chicken Populations Divergently Selected for Body Weight Compared to a Commercial Broiler

**Figure 4:**
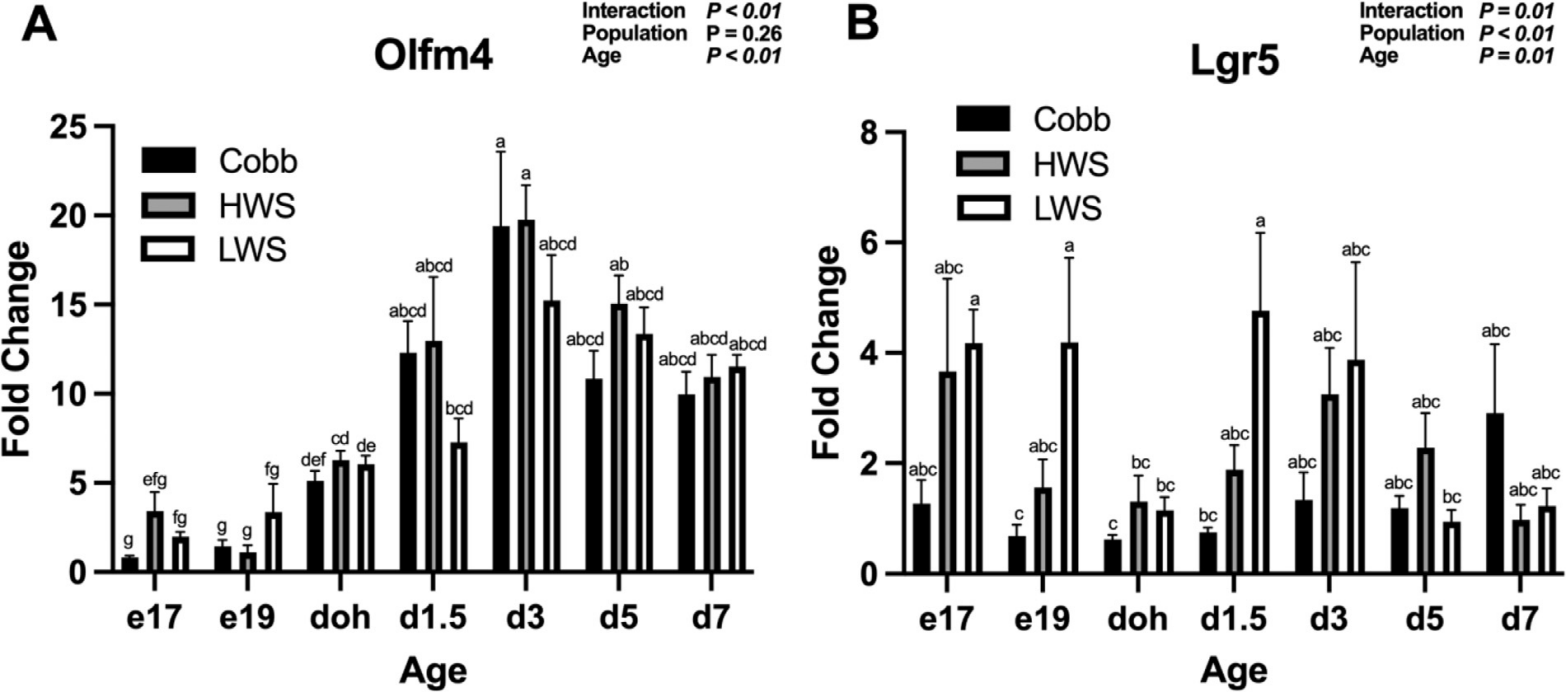
Gene expression of stem cell markers in the jejunum of chicken populations divergently selected for body weight compared to a commercial broiler. Relative mRNA abundance of (A) Olfactomedin 4 (Olfm4) and (B) Leucine-rich repeat containing G protein-coupled receptor 5 (Lgr5) is shown. Sections of jejunum were collected on embryonic d 17 (e17), embryonic d 19 (e19), d of hatch (doh), d 1.5 (d1.5), 3 (d3), 5 (d5) and 7 (d7) posthatch. Populations included Cobb500 (**Cobb**), high weight select (**HWS**), and low weigh select (**LWS**). Statistical significance was determined using 2-factor ANOVA and Tukey HSD mean separation. Each value represents the back-transformed means from 6 to 8 embryos or chicks. ^a-g^Bars with different letters are significantly different (*P* < 0.05).

**Figure 5:**
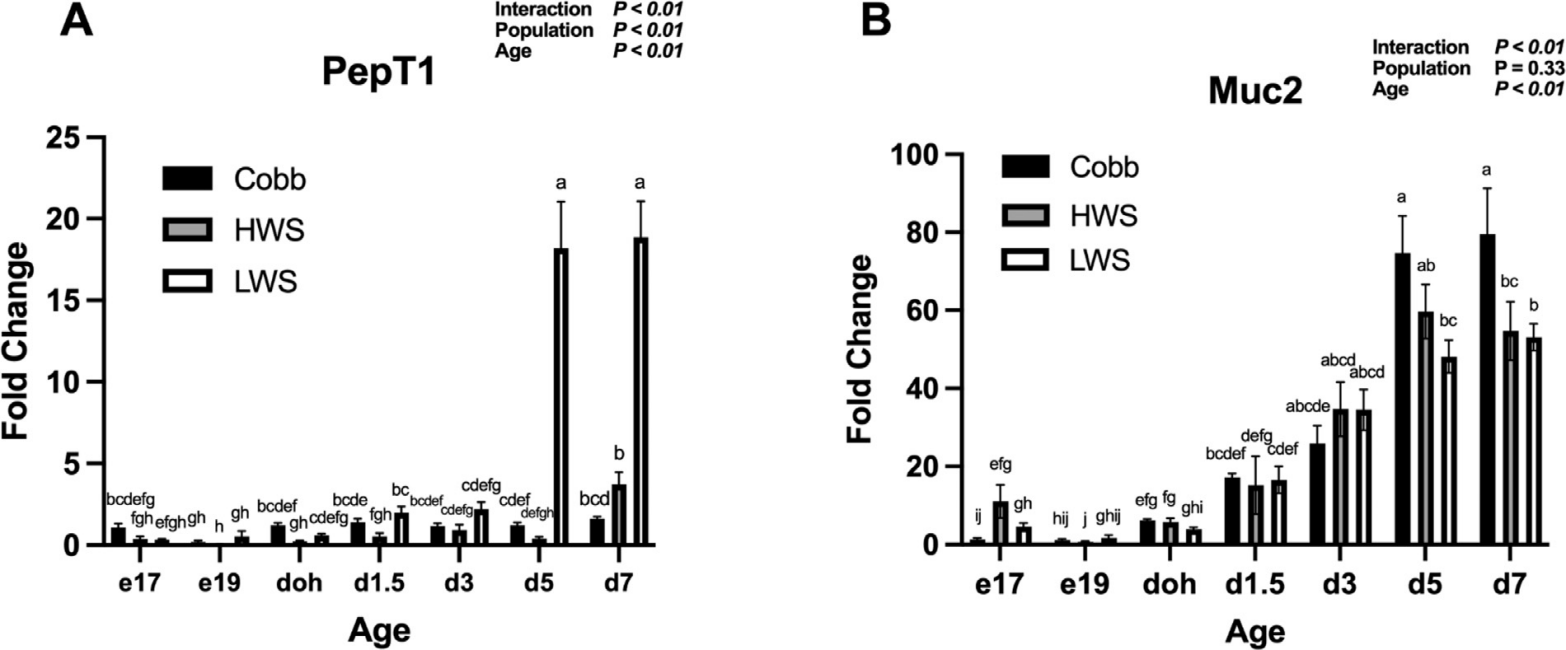
Gene expression of PepT1 and Muc2 mRNA in the jejunum of chicken populations divergently selected for body weight compared to a commercial broiler. Relative mRNA abundance of (A) Peptide transporter 1 (PepT1) and (B) goblet cell marker Mucin 2 (Muc2) is shown. Sections of jejunum were collected on embryonic d 17 (e17), embryonic d 19 (e19), d of hatch (doh), d 1.5 (d1.5), 3 (d3), 5 (d5) and 7 (d7) posthatch. Populations included Cobb500 (**Cobb**), high weight select (**HWS**), and low weight select (**LWS**). Statistical significance was determined using 2-factor ANOVA and Tukey HSD mean separation. Each value represents the back-transformed means from 6 to 8 embryos or chicks. ^a-j^Bars with different letters are significantly different (*P* < 0.05).

**Figure 6:**
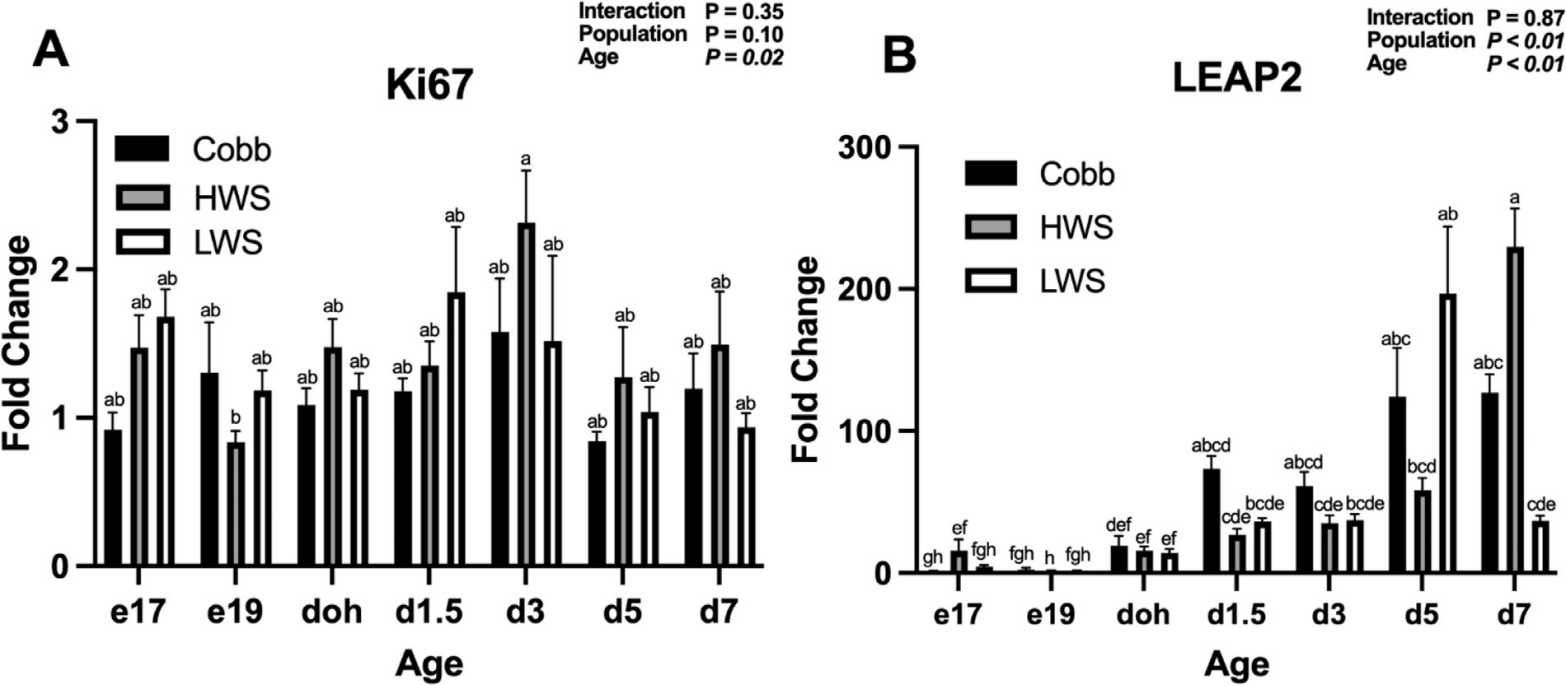
Gene expression of Ki67 and LEAP2 mRNA the jejunum of chicken populations divergently selected for body weight compared to a commercial broiler. Relative mRNA abundance of **(A)** Marker of Proliferation (Ki67) and **(B)** Liver-enriched antimicrobial peptide 2 (LEAP2) is shown. Sections of jejunum were collected on embryonic d 17 (e17), embryonic d 19 (e19), d of hatch (**doh**), d 1.5 (d1.5), 3 (d3), 5 (d5) and 7 (d7) posthatch. Populations included Cobb500 (**Cobb**), high weight select (**HWS**), and low weight select (**LWS**). Statistical significance was determined using 2-factor ANOVA and Tukey HSD mean separation. Each value represents the back-transformed means from 6 to 8 embryos or chicks. ^a-e^Bars with different letters are significantly different (*P* < 0.05).

For Olfm4 mRNA, there was a Population × Age interaction (*P* < 0.01; Figure 4A). Olfm4 mRNA in Cobb and HWS increased from e19 to doh (*P* < 0.05), doh to d3 (*P* < 0.001), and then remained the same from d3 to d7 (*P* > 0.05). Expression in LWS increased from e19 to doh (*P* = 0.014), but then was similar at ages doh to d7 (*P* > 0.05). For Lgr5 mRNA, there was a Population × Age interaction (*P* < 0.01; Figure 4B). Lgr5 mRNA was lower in Cobb than LWS on e19 (*P* < 0.05) and d1.5 (*P* < 0.05). At all other ages, no differences in Lgr5 mRNA were observed between populations (*P* > 0.05). For peptide transporter PepT1 mRNA, a Population × Age interaction was observed (*P* < 0.01; Figure 5A). Expression of PepT1 mRNA on d5 and d7 increased 10-fold in LWS compared to Cobb and HWS. No other differences in expression were observed (*P* > 0.05). For Muc2 mRNA, a Population × Age interaction was observed (*P* < 0.01; Figure 5B). On e17, expression of Muc2 mRNA in HWS was greater than Cobb (*P* = 0.0055). From e19 to d3, there were no differences in Muc2 mRNA between populations (*P* > 0.05). At d5, Muc2 mRNA was greater in Cobb than LWS (*P* = 0.031), while at d7, Muc2 mRNA was greater in Cobb than HWS (*P* < 0.0001) and LWS (*P* < 0.0001). For proliferation marker Ki67 mRNA, there was a main effect of Age (*P* = 0.02) with an increase in Ki67 mRNA on d3 from e19 (Figure 6A). For antimicrobial peptide marker LEAP2 mRNA, there were main effects of Population (*P* < 0.01) and Age (*P* < 0.01; Figure 6B). LEAP2 mRNA increased from e17 to d7 and was greater in HWS than Cobb and LWS.

## DISCUSSION

Genetic selection in commercial broilers has enhanced growth rate, and the first week posthatch represents an increasing proportion of the total growing period (Willemsen et al., 2008). Selection over generations for growth performance traits has influenced organ development including the gastrointestinal tract and its enzymes that aid in nutrient digestion during absorption (Nitsan et al., 1991). Early times in the lives of chicks represents an important period relative to optimizing performance through digestive efficiency. In this study we compared high and low weight lines, which are closed pedigree populations of White Plymouth Rocks that are part of a well-documented long-term selection experiment for the single trait BW and 8 wk of age, (Siegel, 1962; Siegel, 2014; Siegel et al., 2019, Siegel, 2023; Harrison et al., 2023). In contrast, the Cobb 500 is a composite of several meat lines mated to produce a cross between 2 F_1_s for its commercial product (Cobb 500, 2022). Thus, we were interested in examining changes in intestinal morphology and gene expression in these populations that have been subjected to different selection criteria.

Cobb were heavier than the HWS and LWS and the differences in body weight can be partially attributed to the founder populations. HWS and LWS were descendants of White Plymouth Rock chickens, dual-purpose chickens valued for their meat and egg-laying abilities. Cobb are used in modern production for meat and are a hybrid involving breeds to capitalize on growth performance and breast meat deposition. LWS were similar in body weight to HWS on e19 when metabolism is switched from fatty acid oxidation to glucose metabolism (Givisiez et al., 2020). This effect could be a result of yolk absorption differences between these 2 selected populations. HWS have been shown to exhibit decreased lipolysis capabilities (Calabotta et al., 1985), reduced fatty acid oxidation efficiency, and impaired metabolic flexibility (Zhang et al., 2014). This population poorly utilizes yolk nutrients as demonstrated by the greater yolk weight of HWS compared to Cobb and LWS at e17 and e19, which likely lowers growth rate before hatch.

Morphology was used to measure intestinal structure and determine if genetic selection had any effects on villi and crypt depths. Enterocytes on villi absorb nutrients and an increase in villi surface area enhances absorption capacity (Caspary, 1992; Marchewka et al., 2021). An increase in VH after genetic selection for increased body weight was previously observed by Smith et al. (1990). The results of the current study agree with the previous findings, as VH increased on d3 for HWS and d5 for Cobb. Previous studies have shown that growth rate of villi rises around d3 (Uni et al., 1998). The increases in VH in Cobb and HWS represents increased surface area for nutrient absorption compared to LWS and could contribute to greater weight gain, partially explaining the heavier body weights of these populations. Crypts are another major component of intestinal morphology and are defined by stem cells that migrate out of the crypts while differentiating into functional cell types that make up the villi. Crypt depth is thus an indicator of intestinal health, functional status, and denotes efficiency of the epithelial regeneration process of the intestine (Sobolewska et al., 2017). Increased CD was observed in HWS on d3, d5, and d7. An increase in CD is associated with fast tissue turnover and a high demand for new tissue (Umar, 2010). Shorter crypts are associated with decreased tissue turnover, leading to a lower maintenance requirement and higher growth efficiency of the animal as observed in Cobb. Both HWS and Cobb populations have been selected for enhanced growth, but the lengthening of crypts in HWS is representative of increased intestinal turnover and greater energy expenditure. Another indicator of intestinal proficiency is VH/CD. An increase in VH/CD has historically been associated with an improvement in digestion and nutrient absorption (Sobolewska et al., 2017). The greater VH/CD of Cobb on d5 and d7 further support the idea that Cobb have greater intestinal efficiency compared to HWS and LWS.

Expression of genes involved in developing and maintaining the intestinal epithelium were found to be mostly conserved during genetic selection. These genes could be of such fundamental importance for intestinal development and function that even genetic selection cannot alter this process. Stem cells are differentiated towards absorptive cells such as enterocytes for nutrient uptake or secretory cells such as goblet cells (Zhang et al., 2019; Reynolds et al., 2020). Although CD increased in HWS and Cobb, there was no increase in mRNA expression of stem cell markers Olfm4 and Lgr5. Ki67, a marker of cell proliferation that has been previously localized to chicken intestine stem cells (Cloft et al., 2023), also did not change in expression. Proliferation of stem cells involves the division of stem cells into a daughter stem cell and a cell that differentiates into the functional cell types that make up the villi (Fuchs et al., 2004).

Changes in absorptive or secretory cell function determined by PepT1 and Muc2 were also examined to determine if genetic selection of populations affected gene expression. Peptide transporter PepT1 increased dramatically in LWS on d3, d5, and d7. This increase in PepT1 mRNA due to anorexia in LWS was also observed in chicks subjected to feed restriction (Gilbert et al., 2008; Madsen and Wong, 2011) and delayed access to feed (Liu et al., 2020). Downregulation of goblet cell marker Muc2 was also observed in LWS in this study and chicks subjected to delayed access to feed in the study by Liu et al. (2020). In the latter study the decrease in Muc2 mRNA was accompanied with a decrease in intestinal goblet cell density. PepT1 mRNA did not increase in HWS but expression of Muc2 was downregulated, as in LWS. Signaling pathways that differentiate stem cells into secretory cells or absorptive cells that populate the villi are inversely related. If goblet cells denoted by expression of Muc2 are decreased, differentiation of enterocytes could be favored. Therefore, there is a need for additional information on the intestinal epithelium composition and ratio of secretory to absorptive cell types after genetic selection.

Previous studies comparing the LWS and HWS populations have shown that the former display symptoms of anorexia (Zelenka et al., 1998). In chickens, LEAP2 is not only an antimicrobial (Townes et al., 2009) but also an appetite suppressant through interactions with ghrelin (Zheng et al., 2022). Expression of LEAP2 increased on e17 in both LWS and HWS by 25-to-35-fold. Since chicks were still embryos at this timepoint, the increase is likely associated with antimicrobial activity rather than appetite. The decrease of LEAP2 mRNA observed in HWS on d 1.5, 3, and 5 may have contributed to the compulsive eating habits that have been exhibited by this population in previous studies (Liu et al., 2019). This suggests that the intestinal epithelial composition has genetic potential through selection for appetite. Expression of LEAP2 in LWS was similar to Cobb throughout this experiment and did not appear to contribute to the decreased appetite of LWS.

Analysis of intestinal gene expression and intestinal morphology in divergently selected populations compared to a modern Cobb broiler revealed the developmental processes that have been conserved or altered with genetic selection. Some genes such as Olfm4, Lgr5, and Ki67 were conserved across populations and are not influenced by selection for body weight, whereas PepT1 was greatly increased following selection for low body weight.
